# Sustainable activated carbon from copper pod tree leaves for efficient tetracycline removal and regeneration

**DOI:** 10.1038/s41598-025-02213-6

**Published:** 2025-05-19

**Authors:** Hari Om Singh, Gokulakrishnan Murugesan, Raja Selvaraj, Thivaharan Varadavenkatesan, Ramesh Vinayagam

**Affiliations:** 1 Department of Chemical Engineering, Manipal Institute of Technology, Manipal Academy of Higher Education, Manipal, 576104 Karnataka India; 2 Department of Biotechnology, M.S.Ramaiah Institute of Technology, Bengaluru, 560054 Karnataka India; 3 Department of Biotechnology, Manipal Institute of Technology, Manipal Academy of Higher Education, Manipal, 576104 Karnataka India

**Keywords:** *Peltophorum pterocarpum*, Tetracycline removal, Sustainable activated carbon, Adsorption isotherm, Adsorption kinetics, Water matrices, Chemistry, Chemical engineering, Environmental chemistry, Green chemistry, Materials chemistry

## Abstract

The persistence of tetracycline (TC) in water poses environmental risks, including antibiotic resistance, necessitating effective removal. This study explores the synthesis and application of activated carbon from copper pod tree leaves for TC adsorption. The adsorbent was produced through orthophosphoric acid activation at low temperature and characterized using multiple techniques. FESEM revealed a porous structure favorable for adsorption, while EDS confirmed the presence of carbon, oxygen, and phosphorus. FTIR identified hydroxyl and carbonyl groups facilitating hydrogen bonding with TC, enhancing adsorption. XRD confirmed the adsorbent’s amorphous nature, while BET revealed a high surface area (865.06 m²/g). XPS further identified C–O and C = O bonds, further supporting adsorption. Kinetic experiments showed that the adsorption fitted to pseudo-second-order kinetics (R² = 0.9765), indicating chemisorption as the dominant mechanism. The isotherm modeling results indicated that the Langmuir model provided an excellent fit to the experimental data (R² = 0.9952), demonstrating a high monolayer adsorption capacity of 103.32 mg/g. Thermodynamics confirmed spontaneity and endothermicity with a ΔH° of 50.75 kJ/mol. The prepared adsorbent effectively removed TC across natural water matrices, retaining high performance after five regeneration cycles.

**Supplementary Information:**

The online version contains supplementary material available at 10.1038/s41598-025-02213-6.

## Introduction

Tetracycline (TC), one of the broad-spectrum antibiotics, revolutionized medication due to its effectiveness, affordability, and versatility in treating various infections^1^. However, its widespread use in human and veterinary medicine, along with agriculture, has inadvertently contributed to the rapid rise of antibiotic resistance, creating serious public health challenges. This challenge is exacerbated by the persistence of TC residues in water, primarily from hospitals and pharmaceutical industries, which are difficult to remove due to technological limitations and environmental factors^2^. This contamination poses a critical concern for health and ecological regulators^3^.

Conventional wastewater treatment methods, encompassing biological, chemical, and physical processes, struggle to eliminate these residues^4^ fully. Biological treatments, like activated sludge and constructed wetlands, utilize natural microbial processes but often find TC degradation challenging due to its complex chemical structure^5^. Chemical treatments, such as chlorination and advanced oxidation processes, offer a more aggressive breakdown of the antibiotic through chemical reactions. However, these methods have drawbacks; chlorination can produce harmful by-products, while advanced oxidation processes (AOPs) require high energy input, making them less economical^6^. Studies highlight these challenges, showing that conventional wastewater treatment plants only achieve a removal efficiency of 48–77% for TC^7^. Consequently, significant amounts of these antibiotics persist in treated water, posing ongoing environmental contamination concerns and highlighting the urgent need for advancements in treatment methods.

Amongst alternative approaches, adsorption is an effective method that has drawn interest due to its simple process, affordability, and higher efficacy in eliminating impurities from aqueous solutions^8^. Unlike other methods, adsorption does not generate harmful by-products and can effectively target various pollutants, including antibiotics like TC. Adsorption is known to be advantageous for wastewater treatment, where the complexity of the water matrix and the need for highly selective removal are critical. Studying adsorption mechanisms is crucial for optimizing materials and processes that deliver high removal efficiency and environmental sustainability. Physical methods, including the use of activated carbon (AC), offer effective solutions to remove TC from water. Because of the high specific surface area (SSA), tailored surface properties for enhanced adsorption, rapid kinetics, and potential for regeneration and reuse^9^. One key advantage of AC is its ability to be tailored and functionalized with specific surface chemistry. Surface modifications can enhance interactions with TC molecules, improving adsorption selectivity and efficiency. This capability allows AC to target and capture TC contaminants selectively from complex water matrices, minimizing the removal of harmless constituents^10^.

AC has been widely studied for its effectiveness in removing TC from aqueous streams, utilizing various sources such as palm leaves^11^, macroalgae, *Sargassum (sp.)*^12^, and bamboo pulp^13^. However, the preparation of these adsorbents often requires high temperatures ranging from 600 − 800 ℃. Despite these conditions, the resulting adsorption capacities remain relatively low, specifically between 40 and 80 mg/g^14^. Additionally, the SSA reported in various studies for these ACs are moderate, such as 563.93 m²/g^15^, 766.75 m²/g^16^, and 794 m²/g^17^, which can further limit their adsorption potential. *Peltophorum pterocarpum* tree, commonly known as the copper pod tree, is widely appreciated as a shade tree for its dense, spreading crown and is commonly found in Southeast Asia. This tree is broadly recognized for its ornamental value. Copper pod trees are abundantly available throughout India, presenting an opportunity to utilize this sustainable biomass as an economical feedstock for AC preparation. Earlier studies have reported the preparation of copper pod tree leaf AC often requires high temperatures ranging from 400 − 600 ℃ and lower SSA like 409.01 m²/g^18^ and 443 m²/g^19^, which may restrict their effectiveness. To date, there are currently no published reports regarding the adsorption of TC using AC prepared from copper pod tree leaves. Considering these facts, our study adopts a low-temperature method for preparing AC, to enhance the adsorption capacity for TC removal. This approach aims to overcome the limitations identified in previous studies, providing a more efficient and sustainable alternative for contaminant removal.

Moreover, this study addresses the challenges of existing biomass-derived ACs, including high costs, complicated synthesis, low specific surface area, and limited adsorption potential. Using copper pod tree leaves as a sustainable material and H_3_PO_4_ as the activating agent, our approach proposes a cost-efficient and sustainable alternative. The use of copper pod tree leaves provides an abundant, low-cost raw material, while H_3_PO_4_ simplifies the activation process, being safer and less corrosive than other activators like H_2_SO_4_, ZnCl_2_, K_2_CO_3_, KOH, and HCl^20^. In contrast to these alternatives, which require high activation temperatures (500–900 °C) and inert atmospheres, our method operates efficiently at 400 °C for 2 h in ambient conditions, reducing energy consumption and safety risks. This process produces high-surface-area AC with enhanced adsorption capacity, making it highly effective for TC removal from water. The low toxicity of H_3_PO_4_ and a neutralization step further enhance sustainability, making this method a scalable, eco-friendly solution for wastewater treatment. The low toxicity of H_3_PO_4_ and the neutralization step further enhance sustainability, making this method a scalable and eco-friendly solution for wastewater treatment.

Therefore, this study reports the AC synthesis from copper pod tree leaves and its effectiveness in adsorbing TC. Advanced characterization techniques were employed to analyze the adsorbent properties. Subsequently, the research delves into the TC adsorption properties by examining the influence of temperature, initial concentration of TC, pH of solution, and contact time. Furthermore, kinetic, isotherm, and thermodynamic studies were done to understand the adsorption mechanism.

## Materials and methodologies

### Chemicals and plant source collection

Orthophosphoric acid (OP) and sodium bicarbonate were procured from Merck, India, along with other analytical-grade chemicals. TC hydrochloride (C₂₂H₂₄N₂O₈·HCl, M_w_= 480.90 g mol^− 1^, *λ*_max_ = 276 nm) was procured from Himedia, India. To ensure uniformity throughout the trials, distilled water was utilized. The raw material, copper pod tree leaves, used for preparing AC, was collected from the campus grounds of MIT, Manipal, India.

### Adsorbent Preparation

**Fig. 1:**
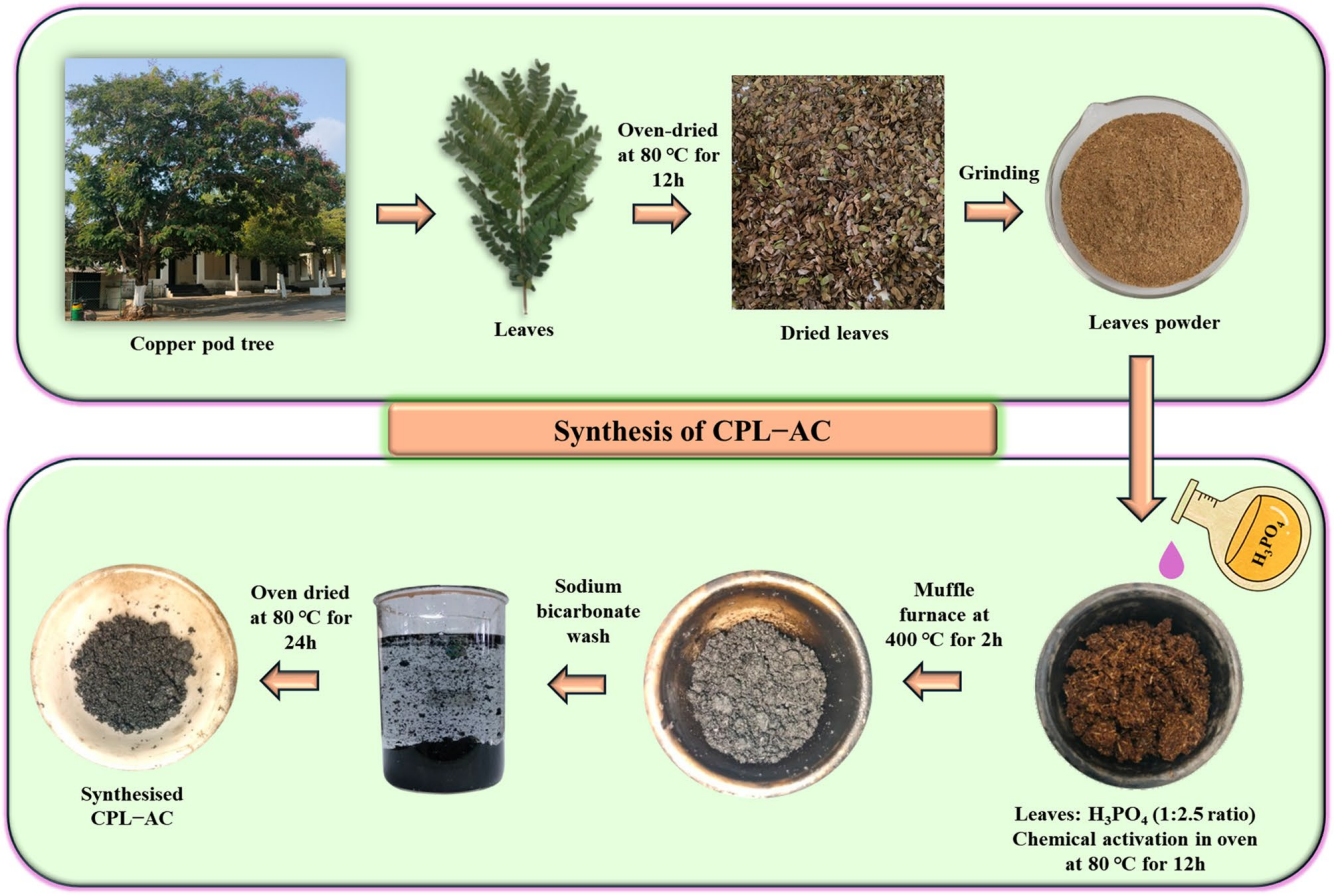
Steps involved in the synthesis of CPL-AC from copper pod tree leaves.

Copper pod tree leaves were collected and washed several times with distilled water to remove contaminants. The leaves were subsequently oven-dried at 80 ℃ and ground into a fine powder. The ground biomass was added with OP in a 1:2.5 ratio and aged for 6 h. Following this, the mixture was oven-dried at 80 ℃ for 12 h. Once dried, the material was subjected to muffle furnace carbonization at 400 ℃ for 2 h. The obtained products were repeatedly washed with a 1% sodium bicarbonate solution until the pH reached 7. Subsequently, the material was dried at 80 ℃ for 24 h, which yielded CPL − AC **(**Fig. 1**)**.

### Characterization of CPL − AC

Field emission scanning electron microscopy (FESEM) was employed to examine the surface appearance of the produced CPL − AC using a Neon 40 instrument (Carl Zeiss, Germany). To further characterize the material and identify its elemental compositions, Energy-dispersive X-ray spectroscopy (EDS) was used with an X-act detector (Oxford Instruments, UK). Functional elements were recognized by Fourier-transform infrared (FTIR) spectroscopy with a Shimadzu – 8400S spectrometer, Japan. The binding energies of the elements were determined via X-ray photoelectron spectroscopy (XPS) using a Thermo Fisher Scientific instrument, UK. The SSA of the material was determined using Smart Instruments India BET equipment by Brunauer–Emmett–Teller method. In addition to these structural and elemental analyses.

### Adsorption experiments

Initial adsorption experiments focused on optimizing the pH for maximum TC adsorption. Using a constant CPL − AC dosage of 0.3 g/L and a TC concentration of 25 mg/L, the pH was varied from 2 to 12 while maintaining a constant temperature of 30 ℃ and a stirring speed of 150 rpm. Subsequently, the effect of the dose on TC removal was assessed at the optimal pH. Varying amounts of CPL − AC, ranging between 0.1 and 0.5 g/L, were introduced to 25 mg/L TC solution of 100 mL, maintaining a constant temperature and stirring speed. Adsorption equilibrium, indicating the completion of the adsorption process, was typically achieved within 180 min, allowing for the analysis of CPL − AC dosage on TC removal efficiency. The temperature influence was then examined by changing from 20 to 50 ℃ while maintaining a constant CPL − AC of 0.3 g/L and 25 mg/L TC concentration. The percentage removal of TC was analyzed after 180 min of contact time with constant stirring at 150 rpm. Finally, the impact of initial TC concentration was examined at the previously determined optimal pH and dosage. TC removal efficiency was monitored at different time intervals (0–180 min) using initial TC concentrations between 10 and 50 mg/L. The experiments were done thrice using a CIS 24 Plus orbital incubator (Remi) to maintain a controlled temperature and agitation speed. At specific time intervals, samples were drawn and centrifuged at 10,000 rpm for 10 min (Eppendorf-5425). The absorbance of the supernatant was then analyzed by a UV–vis spectrophotometer (Shimadzu – 1900i). The mean value of the triplicate measurements was used for analysis.

The correlation between TC removal efficiency (R) and adsorption capacity (q_e_) was determined by employing Eqs. 1 and 2.

1 $$\:R=\frac{{C}_{in}-{C}_{t}}{{C}_{in}}\times\:100\:$$

where, C_in_ and C_t_ (mg/L) denote the initial and the TC concentration at a given time ‘t’, correspondingly.

2 $$\:{q}_{e}=\frac{{C}_{in}-{C}_{e}}{W}\:\times\:{V}_{s}$$

wherein, C_e_​ (mg/L) denotes the equilibrium TC concentration, V_s_ (L) signifies the volume of the solution, and W (g) refers to the quantity of CPL − AC used.

### Adsorption studies

#### Adsorption kinetics

To explore the adsorption kinetics, three kinetic models viz., pseudo-first-order (PFO), pseudo-second-order (PSO), and intraparticle diffusion (IPD)^21^, as denoted by Eqs. (3), (4), and (5), respectively.

3 $$\:\text{P}\text{F}\text{O}\:\text{m}\text{o}\text{d}\text{e}\text{l}:\:{q}_{t}={q}_{e}(1-\text{exp}(-{k}_{1}t))$$

4 $$\:\text{P}\text{S}\text{O}\:\text{m}\text{o}\text{d}\text{e}\text{l}:\:{q}_{t}=\frac{{q}_{e}^{2}{k}_{2}t}{qe{k}_{2}t+1}$$

5 $$\:\text{I}\text{P}\text{D}\:\text{m}\text{o}\text{d}\text{e}\text{l}:\:{q}_{t}={K}_{P}^{0\cdot\:5}+C\:$$

In these models, qt and qe represent the respective adsorption capacities at time t and at equilibrium. Furthermore, k_1_ and k_2_ are the rate constants for the PFO and PSO equations, while K_p_ and C denote the IPD factor and its constant, respectively.

#### Adsorption equilibrium

To determine the most suitable adsorption isotherm model, experimental data were analyzed using Langmuir, Freundlich, and Temkin models, as described by Eqs. (6), (7), (8).

6 $$\:\text{L}\text{a}\text{n}\text{g}\text{m}\text{u}\text{i}\text{r}:\:{q}_{e}=\frac{{q}_{m}{K}_{L}Ce}{(1+{K}_{L}{C}_{e})}$$

7 $$\:\text{F}\text{r}\text{e}\text{u}\text{n}\text{d}\text{l}\text{i}\text{c}\text{h}:\:{q}_{e}={K}_{F}{C}_{e}^{1/n}$$

8 $$\:\text{T}\text{e}\text{m}\text{k}\text{i}\text{n}:\:{q}_{e}={B}_{T}{ln}\left({K}_{t}{C}_{e}\right)$$

The parameters in these models are defined as follows: K_L_ denotes the Langmuir constant (L/mg), which reflects the affinity between the adsorbate and the adsorbent^22^. q_m_ denotes the maximum adsorption capacity (mg/g), indicating the maximum quantity of adsorbate that could be accommodated by the adsorbent. C_e_ represents the equilibrium concentration, which is the adsorbate concentration in the solution when adsorption equilibrium is reached. Furthermore, K_F_ ((mg/g)/(mg/L)^1/n^) is the Freundlich constant, reflecting the adsorption capacity and intensity. The Freundlich exponent, 1/n, provides insights into the adsorption intensity and surface heterogeneity. Lastly, K_t_ represents the Temkin constant (L/mg), relating to the heat of adsorption, and B_T_ (J/mol) is another Temkin constant, often associated with the adsorbent-adsorbate interactions^23^. The best model was selected based on its high coefficient of determination (R^2^, as well as low reduced Chi-square (χ^2^ and sum of squared error (SSE) values^24^.

#### Thermodynamics studies

The thermodynamic characteristics of TC adsorption onto CPL − AC and the experimental data were examined using the Van’t Hoff model, as represented by Eq. (9).

9 $$\:{k}_{T}=\text{e}\text{x}\text{p}\left[\left(\frac{\varDelta\:{S}^{\circ}}{R}\right)-\left(\frac{\varDelta\:{H}^{\circ}}{R}\right)\frac{1}{T}\right]$$

To further explore the thermodynamic characteristics and adsorption mechanism, various thermodynamic factors were determined using experimental data collected at various temperatures. The equilibrium constant, K_T_ (= q_e_/C_e_), (L/g), was determined from the experimental data. Utilizing these K_T_ values, key thermodynamic factors were analyzed, including Gibbs free energy change, ΔG° (= −RT ln K_T_), (kJ/mol), enthalpy change (ΔH°), and entropy change (ΔS°). ΔH° (kJ/mol) provides insights into the heat absorbed or released during adsorption, while ΔS° (J/mol K) reflects changes in entropy or randomness of the system upon adsorption.

### Desorption and regeneration studies

The reusability of CPL − AC was assessed through regeneration experiments. Adsorption was carried out under ideal conditions using 0.3 g/L of adsorbent with a 25 mg/L TC solution at pH 4, for 180 min. After adsorption, the solution was discarded, and 100 mL of absolute methanol as eluent was added, followed by stirring for 2 h in an incubator at 30 ⁰C. The contents were then decanted, washed, and subsequently dried using a hot air oven. The regenerated CPL − AC was utilized again for subsequent studies^25^.

### TC removal in diverse water matrices

To estimate the efficacy of CPL − AC for the removal of TC from diverse water sources, batch adsorption experiments were conducted using several water mediums that include distilled water, tap water, and water samples from the Arabi Falls, Manipal Lake, Swarna River, and a nearby well. All samples were collected using the standard grab sampling technique. These samples were spiked with 25 mg/L TC and a fixed CPL − AC dose of 0.3 g/L. After adjusting the pH to 4, the mixtures were agitated until equilibrium. The percentage of TC removal was then determined for each water matrix to assess the adsorption potential.

## Results and discussions

### Adsorbent characterization

#### Elemental composition and surface structure

**Fig. 2:**
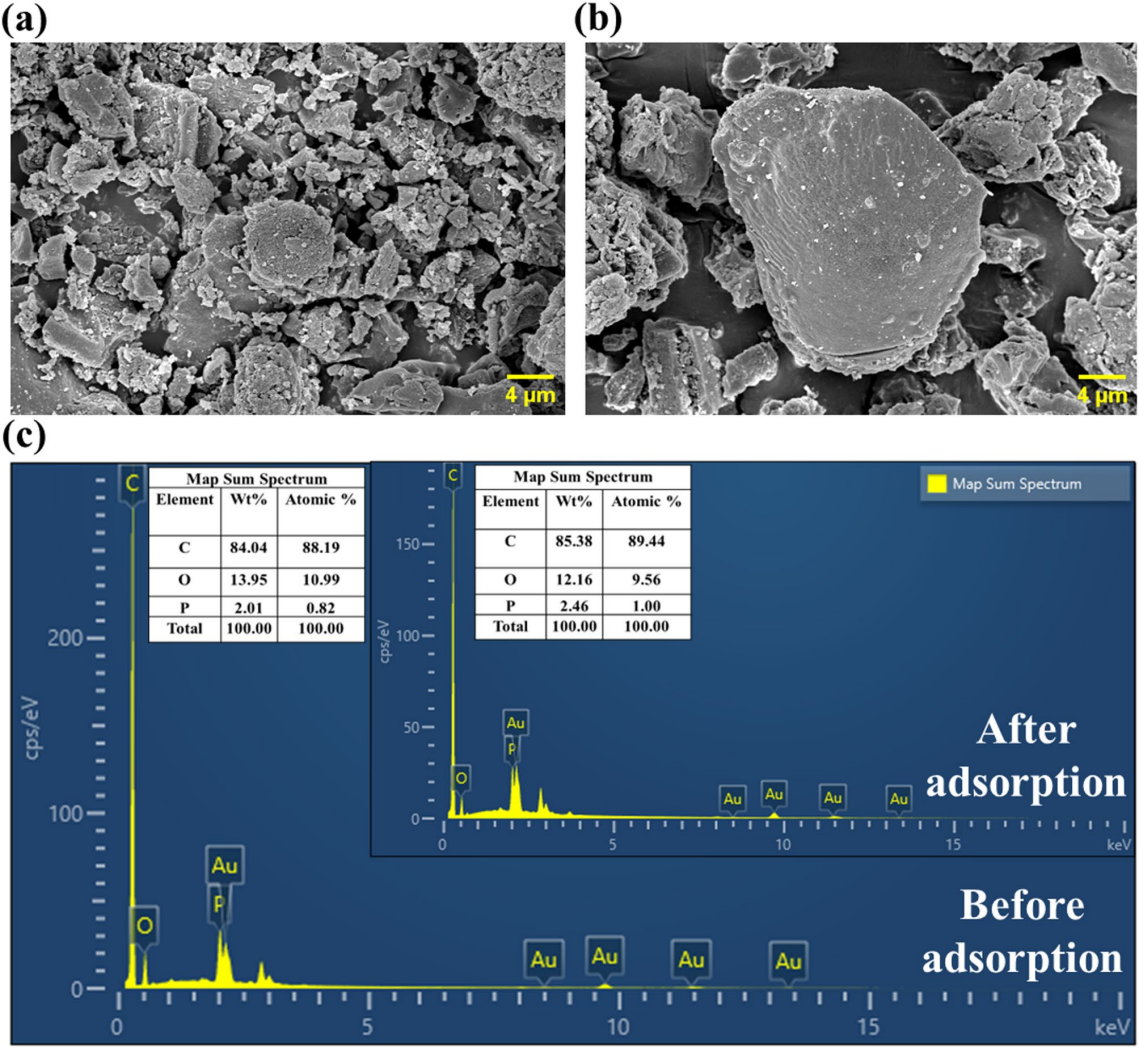
(**a**) SEM image of CPL − AC before, and (**b**) after adsorption (**c**) EDS spectra and elemental mapping before and after adsorption.

The FESEM technique was applied to analyze the structural properties and surface morphology of CPL − AC before and after TC adsorption. As illustrated in Fig. 2a, the surface before adsorption exhibited a rough, porous structure because of thermal carbonization, the AC retained their micro-morphological characteristics^26^, indicating a high surface area suitable for adsorption. BET analysis showed an SSA of 865.06 m²/g, a pore volume of 0.687 cc/g, and a pore size of 3.17 nm, confirming a mesoporous structure^27^. The obtained SSA of CPL − AC is significantly higher compared to other AC adsorbents, like corn stigmata fibers (589 m²/g)^28^ and date seed (376.23 m²/g)^29^. This high SSA, combined with the mesoporous structure, enhances the material’s ability to adsorb TC. The morphological characteristics of AC exhibit a remarkable number of similarities with the AC derived from biomass such as *Cynometra ramiflora* fruit^30^, Olive Stone^31^, *Ulva prolifera*^32^, and semi-coke^4^. After adsorption, the surface became smoother with reduced porosity, suggesting effective pore filling by the adsorbed TC molecules **(**Fig. 2b**)**.

Elemental composition analysis using EDS, as illustrated in Fig. 2c, revealed substantial changes in peak intensities and corresponding composition, corroborating the successful adsorption of TC onto the CPL − AC surface^33^. The most prominent peak in both spectra corresponded to carbon, followed by oxygen and phosphorous (originated from the OP used for the preparation of AC). Also, the gold peaks appear in both spectra due to the gold coating applied to the samples for FESEM imaging to improve conductivity. Post-adsorption, the shifts in peak intensity and composition collectively demonstrate the effective adsorption of TC onto CPL − AC, highlighting its potential as an efficient adsorbent.

#### XRD spectral analysis

**Fig. 3:**
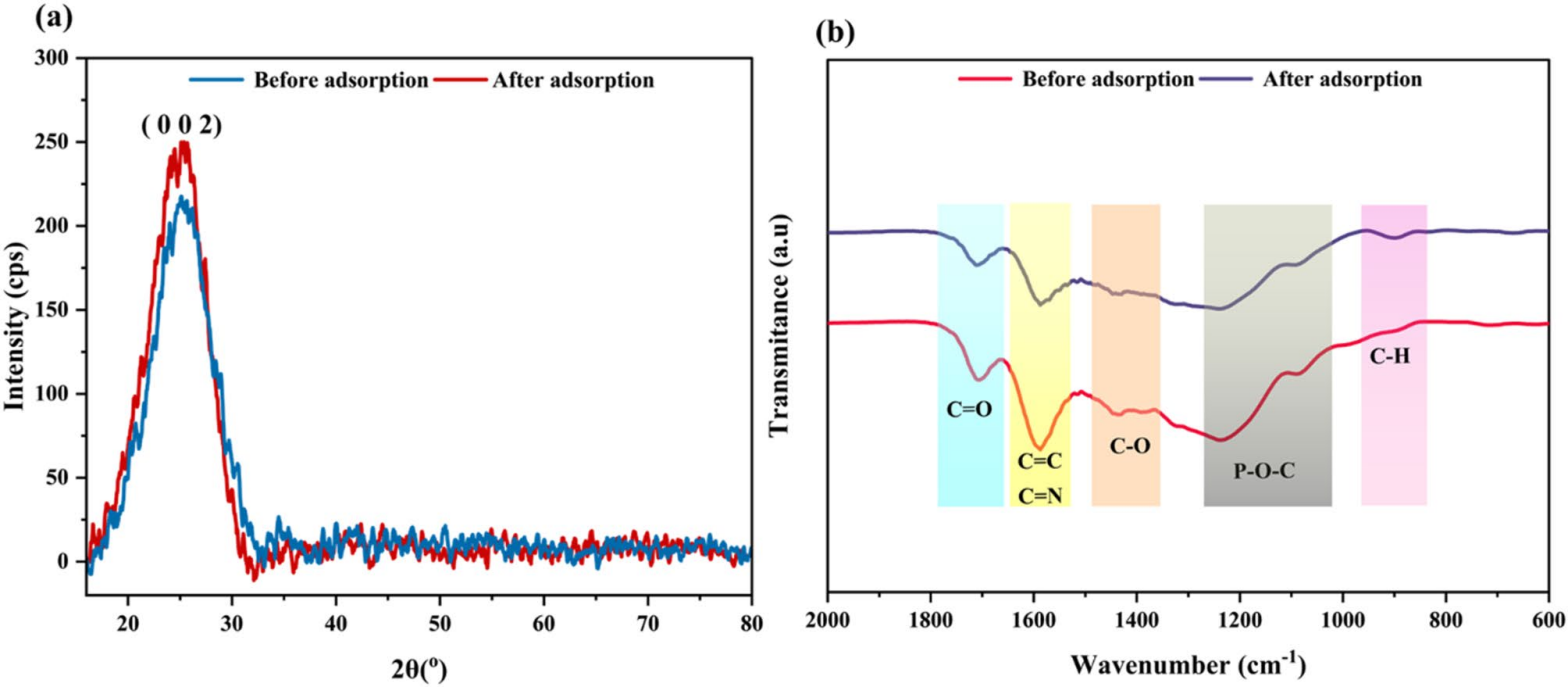
XRD (**a**) and FTIR spectra (**b**) of CPL − AC before and after TC adsorption.

The XRD patterns of CPL − AC, as shown in Fig. 3a, exhibit a prominent diffraction signal at 2θ of 25.39°, related to (002) as mentioned in JCPDS No. 41-1487^34^. The peak indicated the amorphous character of the carbon in CPL − AC, which is more favorable for adsorption because of the irregular pore structure and large surface area. Specifically, while the 2θ values remained consistent before and after adsorption, there is a subtle increase in the observed XRD intensity after adsorption. This increase in intensity can be credited to the rearrangement or alignment of surface functional moieties during the adsorption of TC. However, the absence of any significant shift in the 2θ values suggests that the overall structure of CPL − AC remains unchanged, and adsorption occurs predominantly on the surface without altering the material’s structure. Comparable findings have been reported for AC derived from biomass such as lotus seedpod^35^, and *Syzygium oleana* leaves^36^.

#### FTIR analysis

Using FTIR spectroscopy, the functional moieties on the CPL − AC were analyzed, offering valuable insights into its chemical structure and bonding interactions. The peak at 1705.07 cm^− 1^ indicates C = O stretching vibration, signaling the presence of various carbonyl functional groups. The existence of aromatic structures is confirmed by the C = C stretching vibration at 1587.42 cm^− 1^, characteristic of aromatic rings^37^. This observation is further supported by the C − H bending or C − O stretching vibrations at 1431.18 cm^− 1^, reinforcing the presence of hydrocarbons on the CPL − AC surface^38^. Finally, the peaks at 1238.3 and 1089.78 cm^− 1^ relate to C − O and P − O−C stretching vibrations, suggesting the presence of ether or alcohol groups. These findings are consistent with the activation of CPL − AC by OP, which enhances its surface properties and potential for adsorption^30^.

After adsorption, the FTIR spectrum of CPL − AC shows several notable changes, indicating the interactions between TC and CPL − AC surface. The peak at 1710.86 cm^− 1^, associated with C = O stretching, exhibits a slight shift, suggesting interactions between TC’s carbonyl groups and CPL − AC. This interaction could involve hydrogen bonding or dipole-dipole interactions, further supporting the adsorption mechanism. A shift in the C = C stretching vibrations from 1587.42 to 1570.08 cm^− 1^ suggests interaction among TC and the aromatic rings of CPL − AC. The shift to a lower wavenumber indicates a weakening of the C = C bond, likely due to π-π interactions or hydrogen bonding among TC and the aromatic structure, altering the electron density and vibrational frequency. The 1516.05 cm^− 1^ signal shifted from 1431.18 cm^− 1^, pointing to changes in C-H bending vibrations upon adsorption^38^. This shift implies that TC affects the conformation of aliphatic hydrocarbon chains on the CPL − AC surface. Peaks at 1283.50 and 1093.64 cm^− 1^, previously located at slightly higher wavenumbers, reflect changes in C-O stretching vibrations. These shifts suggest interactions between TC and the ether or alcohol groups on the CPL − AC surface. Finally, a new peak at 904.61 cm^− 1^ in the fingerprint region highlights the development of new bonds or significant alterations in the vibrational modes of the CPL − AC TC complex **(**Fig. 3b**)**. This peak points to intricate molecular interactions occurring during adsorption, reflecting the intricate features of the TC adsorption^39^.

#### XPS results

**Fig. 4:**
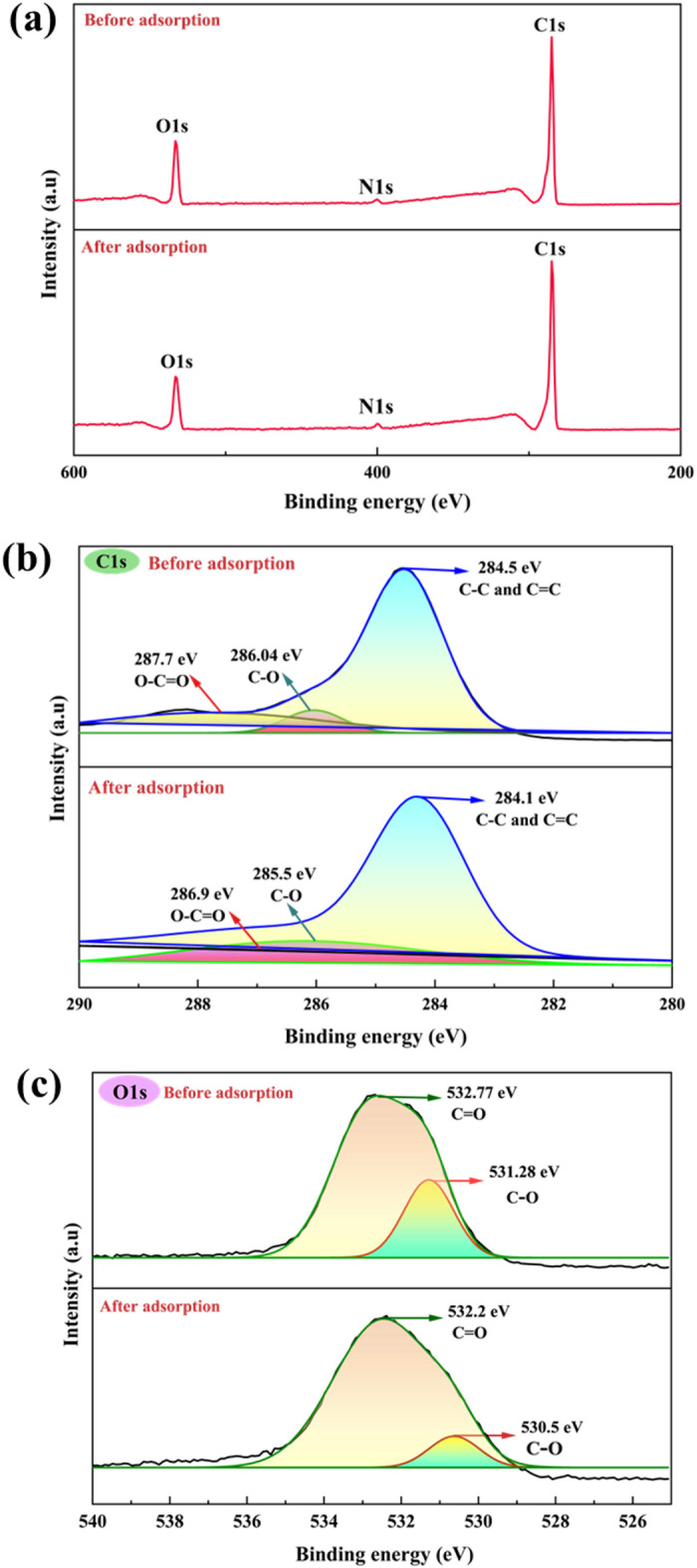
(**a**) XPS compositional spectrum of CPL − AC, along with high-resolution spectra of (**b**) C 1s, (**c**) O 1s.

The XPS spectra (Fig. 4a) reveal the existence of carbon and oxygen, indicating the successful incorporation of carbon- and oxygen-comprising functional moieties on the CPL − AC surface. The dominant C peak highlights the elevated carbon composition of the material. The C1s spectrum showed a broad signal deconvoluted into three definite peaks (Fig. 4b). These correspond to binding energies of 284.51 eV, indicating unfunctionalized carbon (C–C/ C = C); 286.04 eV, associated with ether or hydroxyl group (R–O–R/ C–O); and 287.79 eV, representing carboxyl group (O–C = O)^40^. After adsorption, these peaks have been shifted to 284.1 eV, 285.5 eV, and 286.9 eV, respectively. This deconvolution provides insights into the surface chemistry of CPL − AC, suggesting the presence of both basic and oxygenated functional groups, which can play a role in adsorption. Similarly, the O1s spectrum exhibited two deconvoluted peaks (Fig. 4c); a broad peak at 532.77 eV attributed to C = O bonds, and another at 531.28 eV, related to C-O bond^41^. Post-adsorption, correspondingly, the O1s peaks shifted to 532.2 and 530.5 eV, relating to C = O and C-O bonds. The elemental atomic composition analysis before adsorption showed 82.5% carbon, 15.2% oxygen, and 1.7% nitrogen. Post-adsorption, the composition slightly changed to 82.6% C, 13.3% O, and 1.7% N. The observed changes in elemental composition after adsorption could be due to selective adsorption of carbon-rich molecules, competitive adsorption where carbon outcompetes oxygen-containing molecules for adsorption sites, or a minor surface reaction involving oxygen displacement by the adsorbate. The EDS results corroborate the XPS findings, reinforcing the presence and shifts of these functional groups and further suggesting their role in the multifaceted adsorption mechanism.

The FTIR findings are further supported by XPS analysis, which offers complementary insights into the surface chemistry of CPL − AC. The functional groups identified in the XPS spectrum closely align with the FTIR spectra, reinforcing the surface chemistry. XPS reveals peaks for C–C/C = C bonds and ether or hydroxyl groups (R–O–R/C–O), which are also reflected in the FTIR results indicating hydroxyl and carbonyl functional groups. The increase in transmittance observed after TC adsorption can be primarily attributed to the reduction in available hydroxyl groups. As these hydroxyl groups interact with TC, the O-H stretching band is altered, leading to an increase in the transmittance after adsorption^42^.

### Batch adsorption studies

#### Effect of pH on TC adsorption

**Fig. 5:**
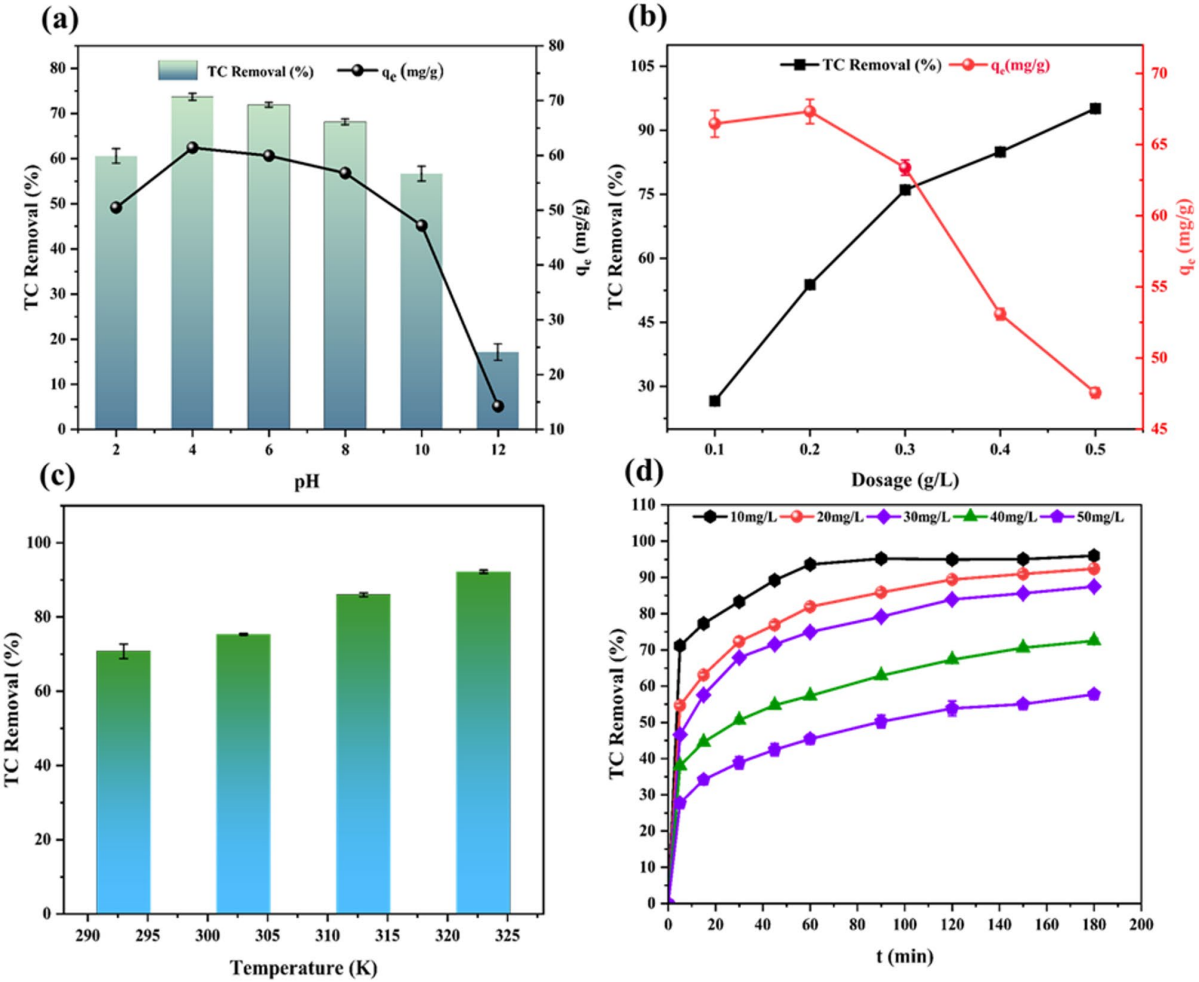
Effect of pH (**a**), Effect of dosage (**b**), Effect of temperature (**c**) and Effect of time and initial concentration (**d**) of TC adsorption onto CPL − AC.

The adsorption of TC onto CPL − AC demonstrates a clear pH dependence, influenced by both the pK_a_ of TC which is 3.3, and the point of zero charge (pH_zpc_) of the AC measured as 6.99 (Fig. 1S). At low pH values, specifically at pH 2, TC exists primarily in its cationic form (pH < pKa), while the CPL − AC surface is positively charged (pH < pH_zpc_). Consequently, an electrostatic repulsion occurs between charged TC moieties and the CPL − AC surface. This electrostatic repulsion results in relatively lower adsorption (60.57%). As the pH rises towards the pH_zpc,_ specifically at pH 4 and 6, the positive charges of the adsorbent surfaces decline, heading to less repulsion and a rise in adsorption, reaching a maximum removal efficiency of 73.67% and 71.91% **(**Fig. 5a**)**.

In addition, the maximum adsorption occurs at pH 4, where TC is predominantly in its neutral form. This indicates that while electrostatic interactions become less significant as the surface charge diminishes, non-electrostatic interactions, like hydrogen bonds and π-π interactions, significantly enhance TC adsorption^16^. These interactions contribute to strong adsorption even under conditions where electrostatic forces alone would be insufficient. At higher pH values (pH 8 and above), the adsorbent surface becomes negatively charged. While this could attract the positively charged portion of the zwitterionic TC, the effect is counteracted by the increasing presence of anionic TC molecules^43^. These negatively charged TC experience significant electrostatic repulsion from the negatively charged moieties (O^−^ and COO^−^) on the activated carbon^40^. This interplay of attractive and repulsive forces results in a gradual decline in adsorption as pH increases beyond the pH_zpc_, reaching a low value at pH 12 (17.05% removal) as depicted in Fig. 5a. A similar study reported that pH 4 was the optimal condition for TC removal using highly porous AC derived from pine fruit biomass^2^.

#### Impact of adsorbent dosage

The CPL − AC dose significantly impacts the adsorption behavior, impacting both removal efficiency and adsorption capacity. The increase in dose from 0.1 to 0.5 g/L, is a directly proportional effect on TC removal, with efficiency rising sharply from 26.71 to 95.88% (Fig. 5b**)**. This enhancement is credited to the better availability of functional adsorption sites and greater surface area provided by the higher dosage^44^. However, this positive correlation does not extend to adsorption capacity. Despite the improvement in removal efficiency, the adsorption capacity per unit of CPL − AC decreases from 66.46 mg/g to 47.94 mg/g with increasing dosages. This inverse relationship is due to the saturation of active spots at higher doses. Although higher dosage initially provides more sites for TC adsorption, the excess nanocomposite beyond a certain point leads to a scenario where fewer TC molecules are available per active site, thus reducing the overall adsorption capacity per unit of CPL − AC^45^. An optimum dose of 0.3 g/L was determined, effectively balancing the removal and adsorption potential. At this dosage, a removal efficiency of 76.66% is achieved while maintaining a reasonable adsorption capacity of 63.37 mg/g (Fig. 5b). This balance ensures efficient utilization of the CPL − AC material by maximizing both available surface area and the binding capacity of each active site without oversaturation. Furthermore, the observation that adsorption equilibrium is reached after 3 h, irrespective of the dosage used, further supports the identification of 0.3 g/L as the ideal dosage for this study^11^. A comparable dosage-dependent trend was observed in a previous study using AC derived from *Auricularia auricula* dregs^46^, and *Caulis spatholobi* residue^47^.

#### Influence of temperature

The temperature effect on TC removal is shown in Fig. 5c which reveals a clear trend that the percentage removal of TC by CPL − AC improves with temperature, indicating an endothermic adsorption process. At 293 K, the removal efficiency was 70.77%, resulting in fewer TC molecules having sufficient energy to overcome the activation energy barrier for adsorption^48^. Significant improvements in removal efficiency were observed as the temperature increased. At 303 K, the removal efficiency increased to 75.35%, and at 313 K and 323 K, it reached 86.01% and 92.23%, respectively. This enhancement is due to two primary factors. First, the higher kinetic energy at elevated temperatures increases the speed and frequency of interactions among TC molecules and the CPL − AC surface, thereby enhancing the likelihood of successful adsorption. Second, the faster diffusion of TC molecules within the solvent allows them to access more adsorption sites in the CPL − AC structure, further improving the efficiency of the adsorption process. Similar results have been observed for activated carbons produced from macroalgae biomass^49^.

#### Influence of initial concentration of TC on adsorption

The adsorption of TC onto CPL − AC is significantly influenced by both initial concentration and contact time (Fig. 5d**)**. The TC removal efficiency shows a consistent trend across various initial concentrations (10–50 mg/L), characterized by an initial rapid increase followed by a gradual plateau. This pattern is attributed to the abundance of available active sites on the CPL − AC surface at the onset of the process. Within the first 80 min, rapid adsorption occurs due to efficient TC uptake facilitated by these readily available sites. This initial phase is driven by a high mass transfer rate, as TC molecules rapidly move from the large volume of the solution to the CPL − AC surface^17^. However, as the adsorption process progresses beyond 80 min, the gradual saturation of active sites leads to a decrease in the rate of TC removal. The slowdown in removal efficiency is because of the gradual saturation of binding sites on the CPL − AC surface. As these sites become increasingly occupied, the driving force for further adsorption reduces, leading to a slower rate of adsorption^42^.

Consequently, the rate of TC removal gradually declines until it reaches a plateau approximately at 180 min, indicating the attainment of adsorption equilibrium (Fig. 5c). At this point, the number of TC molecules being adsorbed onto the CPL − AC is balanced by the number of molecules desorbing back into the solution. This later stage of adsorption, characterized by a slower rate, can be accredited to pore diffusion, where the TC moieties diffuse through the pores of the CPL − AC to reach internal adsorption sites^50^. The rise in initial TC concentration enhances the mass transfer driving force and diffusion rate of TC moieties, resulting in a higher adsorption rate^51^.

#### Kinetic modelling

**Fig. 6:**
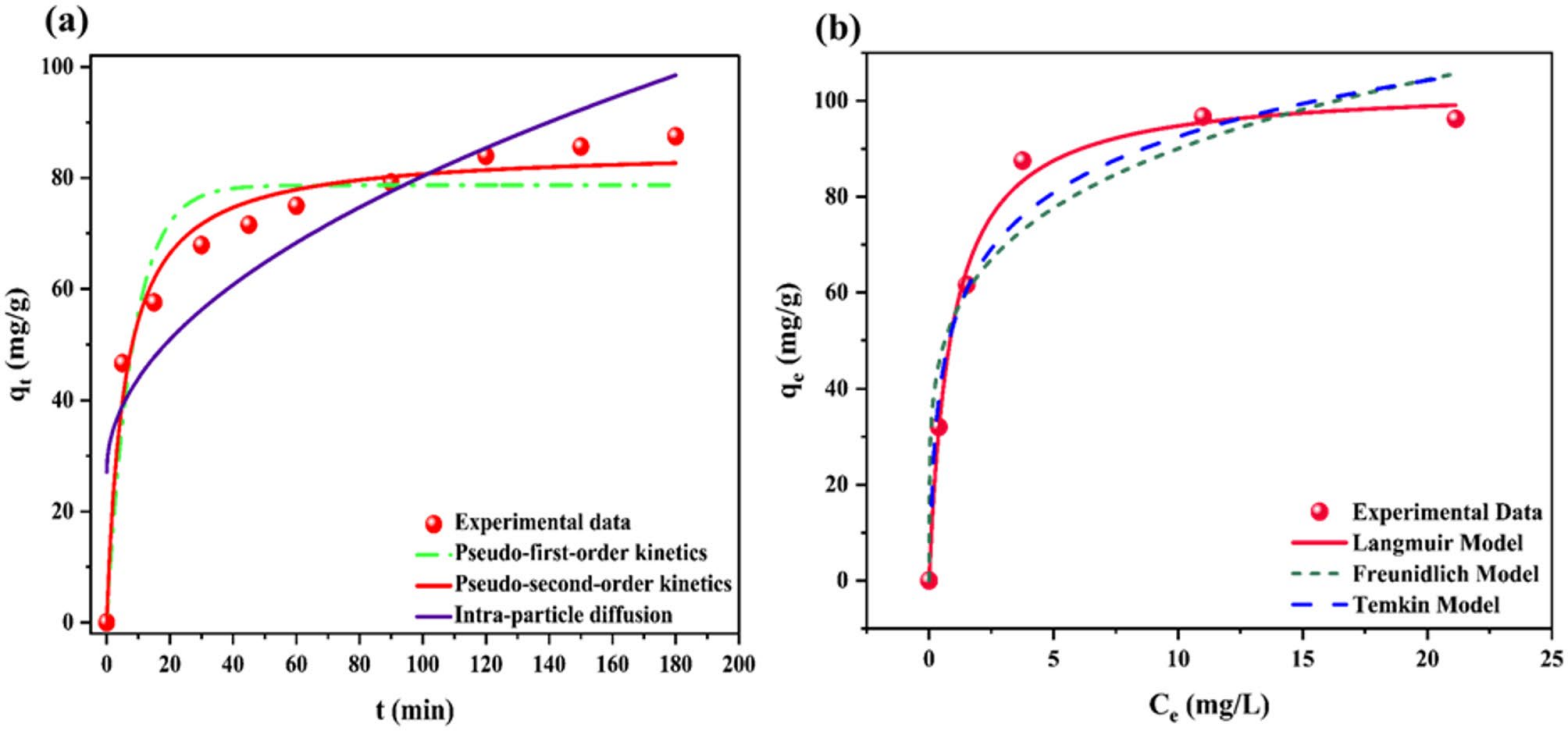
Kinetic studies (**a**), isotherm models (**b**) for the adsorption of TC onto CPL − AC.

To elucidate the kinetics of TC adsorption onto CPL − AC, a comprehensive study employing PFO, PSO, and IPD models was used. Analyzing the fit of experimental data to these models allows for determining the dominant adsorption mechanism, the material’s adsorption capacity (q_e_), and potential rate-limiting steps. The corresponding kinetic plots and the calculated parameters are shown in Fig. 6a; Table 1.

**Table 1:** Kinetic models and isotherm models for TC adsorption onto CPL − AC.

Name of the Models	Parameters	Values
Kinetic Models		
Pseudo First Order	$\:{k}_{1}\left({min}^{-1}\right)$	0.12278
	$\:{q}_{e}$ (mg/g)	78.68
	$\:{R}^{2}$	0.9238
	χ²	59.59
	SSE	476.74
Pseudo Second Order	$\:{\:\:\:K}_{2\:\:\:}$(g/mg.min)	0.00206
	$\:{q}_{e}\:$(mg/g)	85.27
	$\:{R}^{2}$	0.9765
	χ^2^	18.30
	SSE	146.45
Intraparticle Diffusion	K_P_ ((mg/g) min ^0.5^)	5.32047
	R^2^	0.7895
	χ^2^	164.56
	SSE	1316.52
Isotherm models		
Langmuir	q_m_ (mg/g)	103.32
	K_L_ (L/mg)	1.1
	$\:{R}^{2}$	0.9952
	χ^2^	9.29162
	SSE	37.16
Freundlich	K_F_ ((mg/g)/(L/mg)^1/n^	54.97
	1/n	0.214
	$\:{R}^{2}$	0.9358
	χ^2^	124.63
	SSE	498.52
Temkin	B_T_ (J/mol)	16.9
	K_t_ (L/mg)	23.84
	$\:{R}^{2}$	0.917
	χ^2^	85.84
	SSE	257.52

In this case, the equilibrium adsorption capacity (q_e_) calculated by the PSO model, denoted as q_(cal)_, is 85.27 mg/g, which closely aligns with the experimentally found q_e_,_exp_ of 87.48 mg/g. The reduced Chi-square (χ²) value is 18.30, which is significantly lower than those of the PFO and IPD models. The close alignment between the PSO model and observed results is reinforced by a high R^2^ (0.9765) and low SSE value of 146.45, indicating that the PSO kinetics provides the best fit for the adsorption process. This close relation suggests that chemisorption, involving stronger interactions such as covalent or ionic bonding, plays a potential part.

The PFO model, which presumes adsorption primarily through weak van der Waals forces and relies solely on the availability of vacant sites, demonstrated a less accurate fit to the experimental data compared to the PSO. This discrepancy is evident from the calculated q_e_ of 78.68 mg/g, which deviates from the experimentally determined q_e, exp_ of 87.48 mg/g. Furthermore, PFO exhibited a lower R² value of 0.9238, a higher χ² value of 59.59, and a higher SSE value of 476.74, indicating a weaker correlation with the observed data. This implies that the adsorption process does not strictly adhere to first-order kinetics^2^.

The IPD model offers a worthy understanding of the role of pore diffusion in the TC adsorption onto CPL − AC. However, the current analysis indicates that intraparticle diffusion is not the sole rate-limiting factor, as evidenced by its lower R² value of 0.7895. The higher χ² value of 164.56 and SSE value of 1316.52 further highlight the limitations of the IPD model, suggesting it cannot fully describe the adsorption process on its own. A significant factor influencing this process is the boundary layer surrounding the CPL − AC particles, which introduces an additional diffusion barrier.

#### Isotherm modelling

To further investigate the adsorption mechanism, various isotherm model equations, including Langmuir, Freundlich, and Temkin, were used. The non-linear plots of these model equations are illustrated in Fig. 6b, and the corresponding parameter values are shown in Table 1. In this study, the Langmuir model demonstrated the suitable fit, achieving a high R^2^ of 0.9952 and a low SSE of 37.16. The maximum adsorption capacity (q_m_) determined by the Langmuir model was 103.32 mg/g representing the actual adsorption behavior. This supports the hypothesis that adsorption occurs as a monolayer on a homogeneous surface^52^. Notably, as shown in Table 2, recent studies report varying q_m_ for AC; however, the present adsorbent outperforms these, demonstrating superior efficiency for TC removal.

**Table 2:** Comparison of TC adsorption capacity and SSA of various adsorbents.

SL. No	Adsorbent	Preparation conditions	SSA (m^2^/g)	q_m_ (mg/g)	Refs.
1	Waste grape marc activated carbon	HCl, 700 °C,120 min	44.23	17.88	^31^
2	Pine fruit waste magnetic activated carbon	H_2_SO_4,_ 550 °C, 20 min	182.5	43.75	^2^
3	Algal biomass activated carbon	H_3_PO_4_, 400 °C, 2 h	197.53	54.04	^32^
4	Granular activated carbon	–	462.96	4.4	^62^
5	Sawdust magnetic activated carbon composite	Argan gas, 500 °C, 1.5 h	650.4	0.61267	^63^
6	Tea residue activated carbon	KOH, 900 °C, 1 h	747	45.662	^64^
7	Copper pod tree leaves activated carbon	H_3_PO_4_, 400 °C, 2 h	865.06	103.32	This study

The Langmuir constant (K_L_), which represents the affinity between the adsorbent and adsorbate, was found to be 1.1 L/mg, further indicating the adsorbent’s effectiveness in binding TC. Another important parameter derived from the Langmuir model is the separation factor (R_L_ = 1/1 + K_L_C_o_), which provides valuable information on the favorability of the adsorption^53^. An R_L_ value between 0 and 1 indicates favorable adsorption; in this study, the calculated R_L_ of 0.083 falls within the favorable range, further supporting the efficacy of the adsorbent material in removing TC and CPL − AC from the solution^54^.

This heterogeneity of the CPL − AC is reflected in the Freundlich constant (K_F_), which quantifies the adsorbent’s affinity for the adsorbate at a specific concentration. Further insights into the adsorption process are provided by the heterogeneity factor (n), calculated to be 4.67. A value > 1, as observed here, suggests that the adsorption is primarily physically driven rather than chemically driven^11^. The degree of heterogeneity on the adsorbent surface is inversely reflected in the heterogeneity factor (n), with its reciprocal, 1/n (0.214 in this case), providing an identifiable indication. Yet it’s close to 0, this value suggests a surface heterogeneity on the CPL − AC, supporting the suitability of the Freundlich model. Generally, a 1/n value closer to zero indicates more significant heterogeneity^55^. Although the Freundlich model achieved a slightly lower R² of 0.9358 and a higher SSE of 498.52 compared to the Langmuir model, it also captures key aspects of the adsorption behavior. This suggests that while the primary adsorption mechanism may align more closely with the monolayer coverage indicated by the Langmuir fit, the Freundlich model remains relevant in explaining the contributions of surface heterogeneity and potential multilayer interactions. Considering both models provides a deeper insight into the adsorption process, acknowledging the dominant mechanism while recognizing the influence of surface heterogeneity.

The Temkin isotherm model, which assumes chemisorption driven by electrostatic forces, proposes that the heat of adsorption declines linearly with the increase in surface area. This means that as more molecules bind to the adsorbent surface, the interactions between them reduce, leading to a reduction in the energy released during adsorption. However, while the data in this study aligns with the concept of decreasing adsorption heat, as evidenced by the positive Temkin constant (B_T_ = 16.9 J/mol) indicating an endothermic process, the relatively low R^2^ value of 0.917 and high SSE value of 257.52 suggests that the Temkin model is not fitting. The equilibrium binding constant (K_t_ = 23.84 L/mg) provides insight into the initial binding strength between TC and the adsorbent, with a higher value indicating a stronger affinity^53^. The relatively low B_T_ value suggests these binding forces might be relatively weak, probably because of the limited number of high-energy binding sites or weak electrostatic interactions. This discrepancy between the observed data and the ideal Temkin model suggests that other factors, such as heterogeneity of surface sites or multilayer adsorption, might be influencing the adsorption process. Therefore, the Freundlich and Langmuir models give a more accurate fit and a more thorough understanding of the TC adsorption^11^.

#### Thermodynamics studies

Thermodynamic parameters help to assess both the feasibility and spontaneity of TC adsorption onto CPL − AC. The ΔG° values were determined from a plot of K_T_ vs. T (Fig. 2S), and the negative ΔG° values obtained for TC adsorption, such as − 5.08, − 5.45, − 5.84, − 7.85, and − 9.87 kJ/mol, denote that the spontaneity of adsorption^56^. The decrease in ΔG° with rising temperature suggests that higher temperatures favor adsorption, likely due to the high SSA of CPL − AC providing further available binding places for adsorption. The ΔH° value (50.75 kJ/mol) confirms the endothermicity, indicating that the system absorbs heat from the surroundings and requires energy input to process^57^. Additionally, the ΔS° value of 187.62 J/mol K suggests a rise in randomness at the solid-liquid interfaces, which further favors the adsorption process by enhancing the disorder of the material^49^. Ultimately, the thermodynamic data indicate that TC adsorption onto CPL − AC is spontaneous, endothermic, and becomes more favorable with increasing temperature, with the positive entropy value reflecting enhanced disorder at the interface.

#### TC removal studies in diverse water matrices

**Fig. 7:**
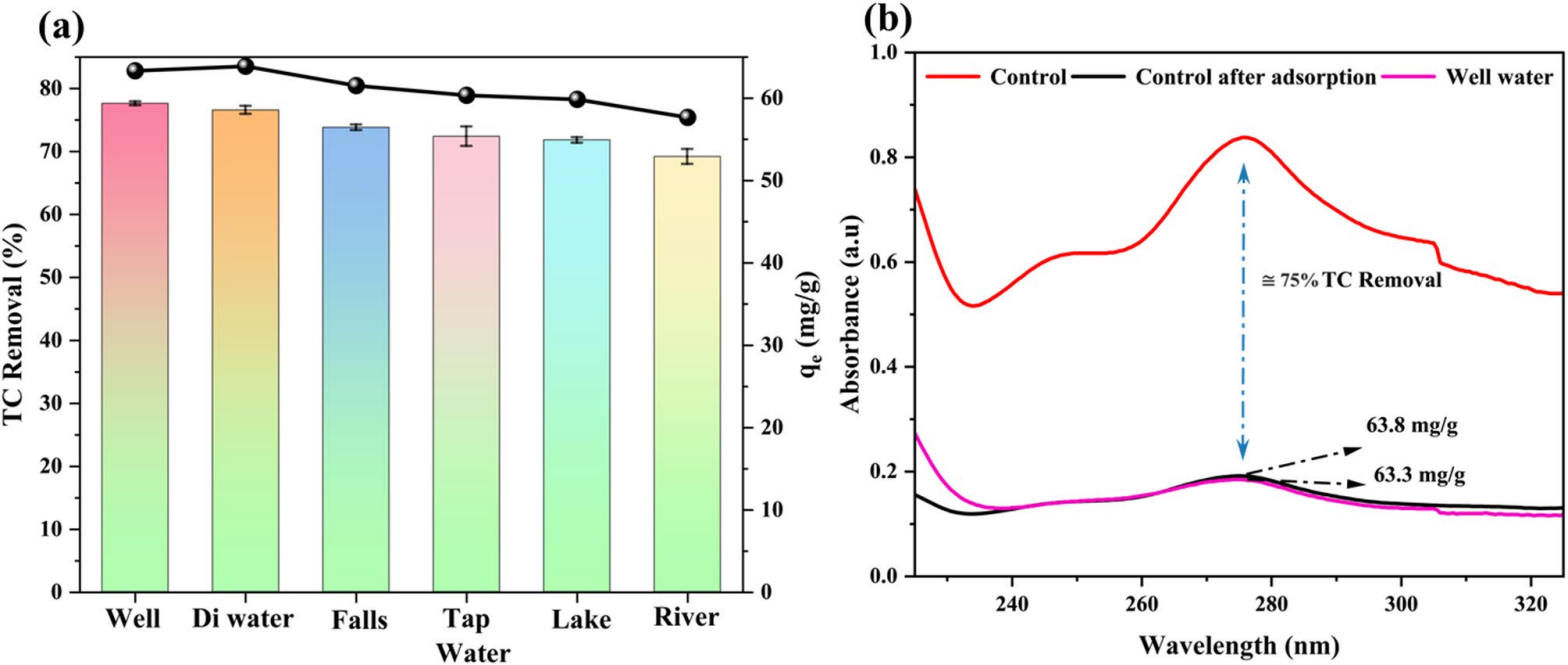
Adsorption of TC onto CPL-AC in simulated natural water matrices (**a**) and spectral analysis of control (DI water) and well water (**b**).

The experiments on simulated TC wastewater were conducted using various water sources, including distilled water (control), well, Arabi Falls, tap water, Manipal Lake, and Swarna River Under the conditions of 0.3 g/L CPL − AC dosage, pH 4, and a 25 mg/L TC concentration, notable removal efficiencies of 76.6%, 77.8%, 73.8%, 72.4%, 71.8%, and 69.9%, respectively, were observed (Fig. 7a). These results demonstrate the strong capability of CPL − AC in removing TC across different water matrices. Additionally, with an optimal dose of 0.3 g/L, an average adsorption capacity of 63.81 mg/g was achieved in natural water samples, which was equivalent to the control sample (63.37 mg/g) (Fig. 7b), further validating the efficacy of adsorbent in both pristine and complex water systems^4,58^. These results highlight the significant potential of adsorbent for real-world applications, particularly in wastewater treatment and the purification of pharmaceutical contaminants from aquatic ecosystems. The high adsorption efficiency, even in the presence of impurities and natural organic matter, indicates that CPL − AC is well-suited for large-scale water treatment processes. Moreover, its effectiveness at minimal dosages makes it a cost-effective and scalable solution for mitigating environmental pollution, particularly in water systems affected by pharmaceutical runoff. The versatility and robustness of CPL − AC make it a promising material for applications in municipal wastewater treatment plants as well as in addressing contamination in natural ecosystems.

#### Desorption and regeneration studies

To assess the reusability of CPL − AC for TC removal, desorption, and reusability tests were done. The regeneration of spent adsorbent is a critical aspect of cost-effective pollutant removal in industrial settings. Therefore, the careful selection of a desorbing agent is paramount to ensure both efficient pollutant removal and minimal degradation of the adsorbent material. As depicted in Fig. 3S, absolute methanol proved to be an effective eluent, achieving a TC removal rate exceeding 73.6% (Control) in the initial cycle. Although this efficiency gradually declined, reaching 45.2% by the fifth cycle, the findings underscore the potential of CPL − AC for multiple-use applications. This gradual reduction in removal efficiency is a common observation in such cyclic processes and can be primarily ascribed to two main reasons. Primarily, with each regeneration cycle, a portion of the adsorption sites on the CPL − AC surface form irreversible bonds with TC, becoming unavailable for subsequent adsorption. This progressive saturation of active sites directly corresponds to the observed decrease in removal efficiency (Fig. 3S). Secondly, repeated exposure to methanol, while essential for desorption, can induce structural changes in the CPL − AC material. Such alterations can negatively impact the adsorption capacity, further contributing to the declining removal efficiency over multiple cycles^59^.

#### TC adsorption mechanism onto CPL − AC

**Fig. 8:**
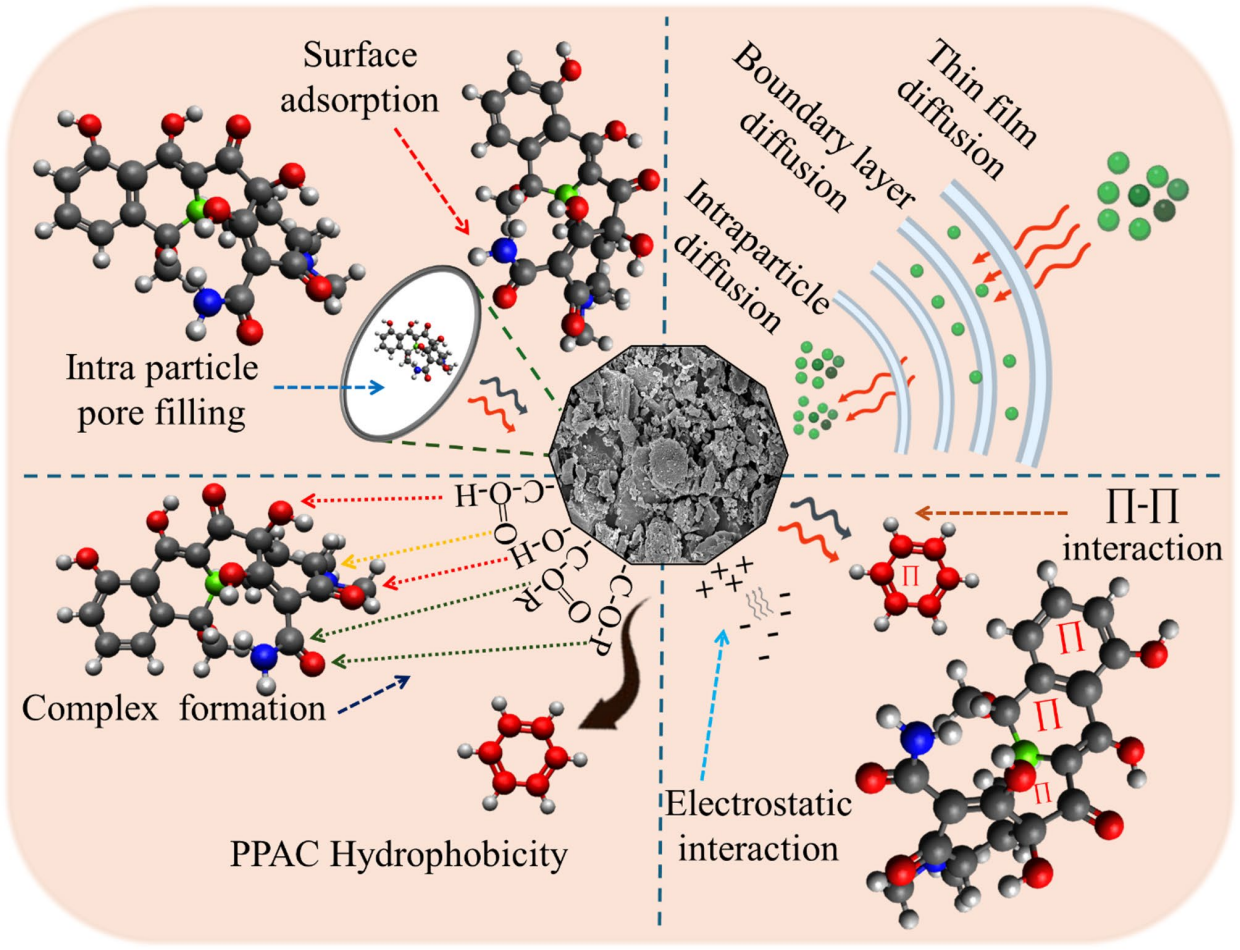
Adsorption mechanism of TC onto CPL − AC (TC structure created using Avogadro 1.2.0; other elements designed using BioRender, Engg, C., 2025).

The adsorption mechanism (Fig. 8) of TC onto CPL − AC is explained by a combination of structural, chemical, and thermodynamic factors^52^. BET analysis reveals that CPL − AC has an exceptionally high SSA, which facilitates the efficient diffusion of TC molecules into the active site of the material. Furthermore, FTIR analysis revealed the occurrence of hydroxyl, carbonyl, and phosphate moieties, which are crucial in facilitating TC adsorption. These functional groups interact with TC primarily through hydrogen bonding, complex formation, electrostatic, and coordination interactions, further enhancing adsorption capacity^38^.

Complementing these findings, XPS analysis reveals the elemental composition and bonding environment on the surface of the adsorbent. The deconvoluted C 1s spectrum indicates various types of carbon bonds, including C − C/C = C, ether/hydroxyl (C − O), and carboxyl (C = O) functional groups, which facilitate π-π interactions and hydrogen bonding with TC. Additionally, the O 1s spectrum supports this interaction by displaying peaks corresponding to C − O and C = O bonding^40^. Additionally, the inherent hydrophobicity of both the adsorbent and adsorbate enhances adsorption affinity. Specifically, aromatic rings in CPL − AC create nonpolar domains that engage in van der Waals interactions with hydrophobic regions of TC^60^.

The adsorption study highlights the pH-dependent nature of the adsorption mechanism. At pH 2, the adsorption capacity is relatively low because of electrostatic repulsions among the positively charged TC molecules and the similarly charged CPL − AC surface. However, as the pH increases to 4, where TC predominantly exists in its neutral form, maximum adsorption is achieved. This transition indicates a reduction in repulsive forces, allowing non-electrostatic phenomena, like π-π stacking and hydrogen bonding, to become more significant. Conversely, at higher pH values, the negatively charged TC molecules are repelled by the negatively charged adsorbent surface, heading to a significant reduction in adsorption^2^.

Kinetic modeling offers deeper insight into the adsorption mechanism, showing that the process closely aligns with the PSO model. This model suggests that chemisorption plays the primary role in the adsorption mechanism, likely facilitated through surface complexation and strong π-π interactions. Reinforcing this, the isotherm study further elucidates the adsorption mechanism, with the Langmuir model providing a better fit for the adsorption data. This supports the predominance of chemisorption, representing monolayer adsorption of TC on the CPL − AC surface through strong binding interactions^61^. Altogether, the combination of structural characteristics and functional group interactions delineates a comprehensive mechanism for the adsorption of TC onto CPL − AC, underscoring its efficacy as a sustainable and efficient adsorbent for TC removal from aqueous solution.

## Conclusions

The tetracycline adsorption onto copper pod tree leaves activated carbon was comprehensively studied in the present study. The prepared activated carbon, features a high specific surface area of 865.06 m²/g and a mesoporous structure, making it highly effective for tetracycline removal from water. The activated carbon was characterized by using FESEM, EDS, FTIR, XRD, BET, and XPS techniques. Optimal adsorption was achieved at pH 4, with a 0.3 g/L dose and a removal efficiency of 76.6%. Kinetic studies exhibited that the PSO model best fit the data, indicating chemisorption, while the Langmuir isotherm model suggested monolayer adsorption with a maximal adsorption capacity of 103.32 mg/g. Thermodynamic analysis confirmed spontaneous and endothermic adsorption. The material also performed well in real-world water matrices, making CPL − AC a promising adsorbent for wastewater treatment. Beyond its adsorption capabilities, the reusability of CPL − AC was also investigated. These observations reveal the capability of CPL − AC as a unique adsorbent, with promising applications for wastewater treatment. The lab-based batch adsorption experiments conducted in the present study may not reflect real wastewater treatment conditions, where unexplored factors like competing ions and organic matter could affect CPL − AC’s efficiency. Its long-term stability in continuous flow systems and industrial feasibility, including cost-effectiveness, scalability, and broader contaminant adsorption, need further study.

## Electronic supplementary material

Below is the link to the electronic supplementary material.

Supplementary Material 1

## Data Availability

The authors declare that the dataset supporting the findings can be found within the article and its accompanying supplementary files.

## Declarations

### Plant Guidelines

The authors confirm that the use of plant/plant parts in the present study complies with international, national, and/or institutional guidelines.

### Permission to collect the plants/plant parts

The tree leaves were taxonomically identified and verified by Dr. Gayathri Pai from the Department of Botany, MGM College, Udupi, India. The tree is widely accessible and unprotected, thus requiring no permits or licenses for harvesting. Furthermore, due to its well-known and prevalent status, a voucher specimen was not submitted to the herbarium.

### Source of the plant used in your study

All plant names and the source of them are in the Methods section.
