# Supplementary material for: Sustainable activated carbon from copper pod tree leaves for efficient tetracycline removal and regeneration
Source: Sci Rep. 2025 May 19;15:17312. doi: 10.1038/s41598-025-02213-6 (PMC12089311; doi:10.1038/s41598-025-02213-6)
Supplement: Supplementary file 1 — Supplementary Material 1 [file 41598_2025_2213_MOESM1_ESM.docx]

**List of Supplementary Figures**

**Figure 1S.** Point-of-zero charge (pH_zpc_) of CPL−AC

**Figure 2S.** Thermodynamic model for the adsorption of TC onto CPL−AC.

**Figure 3S.** Regeneration of CPL-AC using absolute methanol as eluent





**Figure 1S.** Point-of-zero charge (pH_zpc_) of CPL−AC

**
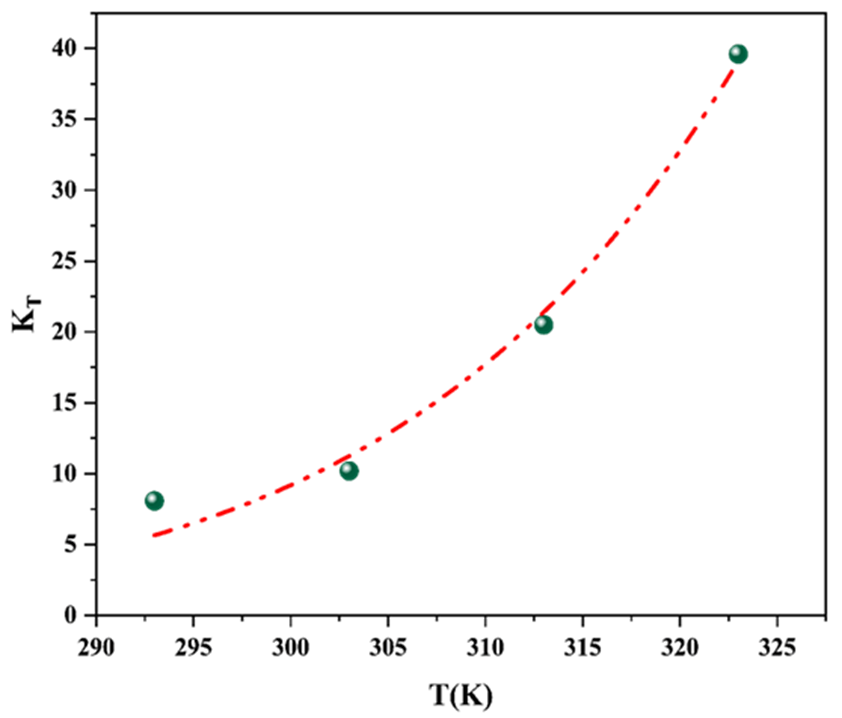
**

**Figure 2S.** Thermodynamic model for the adsorption of TC onto CPL−AC.


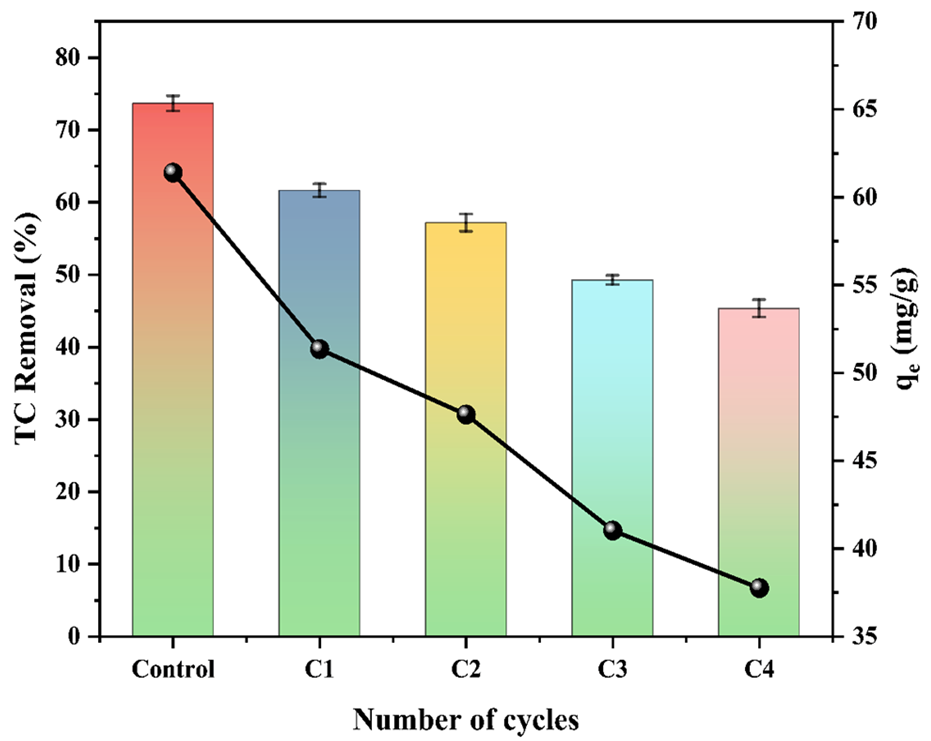


**Figure 3S.** Regeneration of CPL-AC using absolute methanol as eluent
